# Detection and characterization of fungus (*Magnaporthe oryzae pathotype Triticum*) causing wheat blast disease on rain-fed grown wheat (*Triticum aestivum* L.) in Zambia

**DOI:** 10.1371/journal.pone.0238724

**Published:** 2020-09-21

**Authors:** Batiseba Tembo, Rabson M. Mulenga, Suwilanji Sichilima, Kenneth K. M’siska, Moses Mwale, Patrick C. Chikoti, Pawan K. Singh, Xinyao He, Kerry F. Pedley, Gary L. Peterson, Ravi P. Singh, Hans J. Braun

**Affiliations:** 1 Zambia Agricultural Research Institute, Mt. Makulu Central Research Station, Chilanga, Lusaka, Zambia; 2 International Maize and Wheat Improvement Center, Global Wheat Program, CIMMYT, El Batán, Texcoco, México; 3 Foreign Disease-Weed Science Research Unit, United States Department of Agriculture–Agricultural Research Service (USDA-ARS), Ft. Detrick, Maryland, United States of America; Fujian Agriculture and Forestry University, CHINA

## Abstract

Wheat blast caused by *Magnaporthe oryzae* pathotype *Triticum* (MoT) is a threat to wheat production especially in the warmer-humid environments. In Zambia, wheat blast symptoms were observed for the first time on wheat (*Triticum aestivum* L.) grown in experimental plots and five farmers’ fields in Mpika district of Muchinga Province during the 2017–18 rainy season. Infected plants showed the typical wheat blast symptoms with the spike becoming partially or completely bleached with the blackening of the rachis in a short span of time. Incidence of blast symptoms on nearly all wheat heads was high and ranged from 50 to 100%. Examination of diseased plant leaves showed the presence of elliptical, grayish to tan necrotic lesions with dark borders on the leaf often mixed with other foliar diseases. A study was conducted to isolate and identify the causal pathogen(s) using classical and molecular methods and determine the pathogenicity of the detected disease causal agent. Morphobiometrical determination of causal pathogen revealed conidia with characteristic pear shaped 2-septate hyaline spores associated with *M*. *oryzae* species. Preliminary polymerase chain reaction screening of six isolates obtained from wheat blast infected samples with diagnostic primers (MoT3F/R) was conducted at ZARI, Zambia, and subsequent analysis of two isolates with MoT3F/R and C17F/R was performed at USDA-ARS, USA. Both experiments confirmed that MoT is the causal agent of wheat blast in Zambia. Further, pathogenicity tests performed with pure culture isolates from samples WS4 and WS5 produced typical blast symptoms on all the six inoculated wheat genotypes. Results of this study indicate that MoT is causing wheat blast in rain-fed wheat grown in Zambia, thus making it the first report of MoT in Zambia and Africa. This inter-continental movement of the pathogen (disease) has serious implication for wheat production and trade that needs to be urgently addressed.

## Introduction

Wheat blast caused by the ascomycetous fungus *Magnaporthe oryzae* pathotype *Triticum* (MoT) is a serious threat to wheat production [1]. *Magnaporthe oryzae* B.C. Couch (syn. *Pyricularia oryzae* Cavara), is composed of a range of morphologically identical but genetically different host-specific pathotypes that are specialized for infecting rice (*Oryza* pathotype, MoO), wheat (*Triticum* pathotype, MoT), perennial and annual ryegrass (*Lolium* pathotype, MoL), foxtail millet (*Setaria* pathotype) and many other graminaceous hosts. MoT belongs to the *M*. *oryzae* species complex that is known to be pathogenic on a range of hosts, including wheat (*Triticum aestivum* L.), barley (*Hordeum vulgare*), signal grass (*Urocloa brizantha*) and oats (*Arena sativa*) [2, 3]. The disease was first reported in Parana state of Brazil in 1985 [4] and it soon spread to neighboring countries including Bolivia [5], Paraguay [6] and Argentina [7, 8]. Wheat blast became a serious constraint for wheat production in the tropics and sub-tropics regions of Southern Cone of South America. The first appearance of wheat blast beyond South America was reported from Bangladesh in 2016 [9] and has the potential to spread to other major wheat producing areas of India and Pakistan because of the existing favorable conditions for the blast pathogen [10]. Wheat blast infects all aerial plant parts including leaves but is most destructive on spikes by causing shriveled grains of low test weight [11, 12]. Various levels of yield losses due to blast have been reported in many studies but losses of up to 100% are common in areas with favorable environmental conditions [5, 10, 13, 14].

Determination of different pathotypes of *M*. *oryzae* was dependent on pathogenicity tests before the advent of molecular diagnostic assays [1]. Recent progress in the diagnosis of *M*. *oryzae* pathotypes has led to the development of molecular markers that can discriminate between MoT and others [1, 15]. Application of MoT3 primers for example, led to the confirmation of wheat blast in Bangladesh and whole genome sequencing led to relatedness of the Bangladeshi isolates to South American isolates [9, 13]

Optimum temperatures of between 25°C and 30°C coupled with high relative humidity and frequent leaf/spike wetness due to continuous rain, especially if the occurrence of these conditions coincide with heading, favor the development of wheat blast [10, 13]. The aforementioned conditions exist in Zambia only during rainy season. In Zambia, wheat is grown under two agro-climatic systems, rain-fed and irrigated systems. The rain-fed system (usually adopted by small-holder farmers) is characterized by wet, humid and warm conditions while the irrigated system is characterized by cool and dry conditions. Rain-fed wheat production is confounded by frequent outbreaks of spot blotch caused by *Bipolaris sorokiana* (Sacc.) Shoem and Fusarium head blight (FHB) mainly caused by *Fusarium graminearum*, and to a lesser extent loose smut caused by *Ustilago tritici*, powdery mildew caused by *Erisiphe graminis* f. sp. *tritici*, and leaf rust caused by *Puccinia recondita* f. sp. *tritici* among others [16–18].

During the 2017–18 rainy season, an unusually high incidence of white spikes typical of wheat blast symptoms were observed in farmers’ fields and in experimental plots at Malashi in Mpika district of Muchinga Province. Some diseased plants leaves showed the presence of elliptical, grayish to tan necrotic lesions with dark borders on the leaf often mixed with other foliar diseases. The diseased plants displayed symptoms, that is, partial and complete bleached spikes with green canopy closely resembling FHB [19]. However, heads infected with FHB usually have pink or peach fungal spore masses on the spike which were not observed in the diseased spikes detected during 2017–18 season. Additionally, a rapid progression of the blast symptoms was observed in both farmers’ fields and experimental plots during frequent visits of the infected fields. This triggered the suspicion of involvement of pathogen(s) other than *F*. *graminearum*. Such infection was observed in the subsequent two cropping seasons, too, implying the establishment of the disease in the region.

The objectives of this study were therefore to: i) isolate and identify, using classical and molecular methods, the causal pathogens associated with observed blast symptoms on rain-fed wheat, and ii) determine the pathogenicity of the detected disease-causing agent.

## Materials and methods

### Disease incidence, sample collection and pathogen isolation

Disease incidence was estimated in experimental fields at Malashi (altitude 1365.13 meters above sea level (masl), longitude -11.802415, latitude 31.4495) and two farmers’ fields at Mufubushi (one at altitude 1373.7 masl, longitude -12.118651, latitude 31.2409 and the other at 1352.6 masl, longitude -12.100122, latitude 31.2382) in Mpika district of Muchinga Province, Zambia. At each site, 100 plants along two diagonals (50 plants/diagonal) with predetermined sampling points were assessed for blast incidence, which was calculated as a percent proportion of the number of diseased plants out of the 100 sampled. Disease severity was assessed on a scale of 1 to 5 in which 1 = no blast symptoms and 5 = blast symptoms on the whole head. Six suspected wheat blast infected samples were collected from five wheat genotypes (RPWYT-1-35, HON11, HON9, HRWSN80 and Kwale) in experimental fields and one variety, Coucal from farmers’ fields in Mpika district of Muchinga Province. Glumes and seeds were separated and sterilized in 3.5% sodium hypochlorite and thrice rinsed in sterile water. Sterilized glumes and seed of each of the six genotypes were first plated in triplicates (5 -to- 7 samples per 9 cm disposable plates) on agar-agar type 1 (water agar) making a total of 18 plated samples and then incubated at 25°C for 5–7 days. Pear-shaped dual septate hyaline single spores were selected and plated on potato dextrose agar (PDA) amended with Karamycin (1g/L) and incubated for nine days prior to morphological examination with the BA310 microscope. The obtained pure cultures were preserved for subsequent inoculation experiments.

### DNA extraction from fungus

For work at ZARI, Zambia: One representative plate with pure cultures was selected from each of the six triplicated samples (WS1-WS6) and lyophilized overnight at -40°C. The lyophilized cultures were scrapped off the plate into a mortar, to which 1 ml 2% CTAB extraction buffer containing 2% (wt/vol) CTAB, 2% PVP-40, 100 mM Tris-HCl (pH 8.0), 25 mM EDTA, 2M NaCl, and 2% mercaptoethanol (added immediately before use) was added. Using a pestle, the sample was macerated, followed by incubation at 65°C for 15 minutes. An equal volume of chloroform-isoamyl alcohol (24:1) was added to the sample, vortexed and then centrifuged at 12,000 rpm for 15 minutes. The rest of the extraction protocol was conducted as previously described [1]. Dried pellets were suspended in 25 μl DEPC treated water followed by quantification with NanoDROP ONE (Thermal Scientific, CA, USA).

For work at USDA-ARS, USA: A subset of samples WS4 and WS5 was shipped to USDA-ARS FDWSRU under permit from the United States Department of Agriculture–Plant Protection and Quarantine (P526P-19-02423) for further analysis. The two isolates were first single conidia purified, thus obtaining sub-isolates Zambia1.2 (WS4) and Zambia 2.1 (WS5). DNA was extracted from the two sub-isolates and isolates Rb3 (MoO), PL3.1 (MoL), T25 and B2 (MoT) as previously described [1].

### Polymerase chain reaction

At ZARI, Zambia, DNA from the six samples (200–400 ng) was each used in a 25 μl polymerase chain reaction (PCR) reaction volume comprising final concentrations of 1x Dream Taq buffer, 0.2 mM dNTPs, 0.2 μM each of the sense (MoT3F) and antisense (MoT3R) pathotype specific primers [1] and 1 U of Dream Taq DNA polymerase. Thermal cycling conditions included an initial denaturation at 94°C for 2 min, followed by 35 cycles of 94°C for 30 s, adjusted annealing temperature of 55°C for 30 s, and 72°C for 1 min with a final extension step of 72°C for 7 min.

At USDA-ARS, USA, PCR was performed using the reaction conditions described in [1] and [15] with MoT-specific primers MoT3 and C17, respectively. Each reaction was performed using a Mastercycler Gradient Thermocycler (Eppendorf AG, Hamburg, Germany) using 50 ng of DNA per 25 μL reaction with New England Biolabs (NEB) Taq Polymerase (ThermoScientific, San Diego, CA).

PCR products were loaded in 1.2% agarose gel for MoT3 and 2.0% agarose gel for C17 and stained with ethidium bromide (10mg/ml) alongside a 100 bp NEB size marker and electrophoresed at 100 volts followed by visualization with Gel Doc XR System (Bio-Rad Laboratories, Hercules, CA).

### Pathogenicity test on wheat plants, symptom development and pathogen re-isolation

At ZARI, Zambia, pure cultures obtained from six plates that were positive with MoT3 primers were scrapped and washed with 5 ml sterilized water amended with Tween 80 (2 drops/L) as previously described [5]. Spore suspension was diluted 10 times in sterilized water for inoculation. Fifteen seeds of each of the six wheat genotypes replicated three times were sown in peat growth media and later thinned to six plants/pot after germination. Inoculation dates were variable albeit coincided with the heading or flowering dates of respective wheat genotypes. Using 50 ml spore suspension in a 100 ml sprayer with an adjusted nozzle so as to produce a uniform film of water droplets, the fungus was sprayed onto six plants/pot of each wheat genotype. Additionally, six other plants per wheat genotype were inoculated with sterile water amended with Tween 80 to act as control. In order to maintain adequate humidity within the growing environment and support establishment of the fungal spores, all inoculated plants were first watered prior to inoculation and then covered in perforated polythene covers and left for three days before unveiling. The plants were grown in an entry restricted insect-protected screenhouse with a 12-hour day light and mean temperature range of 26.0–33.8°C. Visual inspection of the inoculated plants for blast symptoms commenced 10 days post inoculation (dpi) and continued till maturity.

In order to confirm the veracity of the pathogen identity, glumes and seeds collected from selected symptomatic plants were plated (six/plate) and processed as earlier described. Morphological appearance of the re-isolated spores was compared with the field isolated spores followed by PCR analysis with the MoT3 primers using the same cycling conditions. Amplified fragments of the re-isolated pathogens were electrophoresed and visualized as described above.

## Results

### Field dynamics

**Fig 1:**
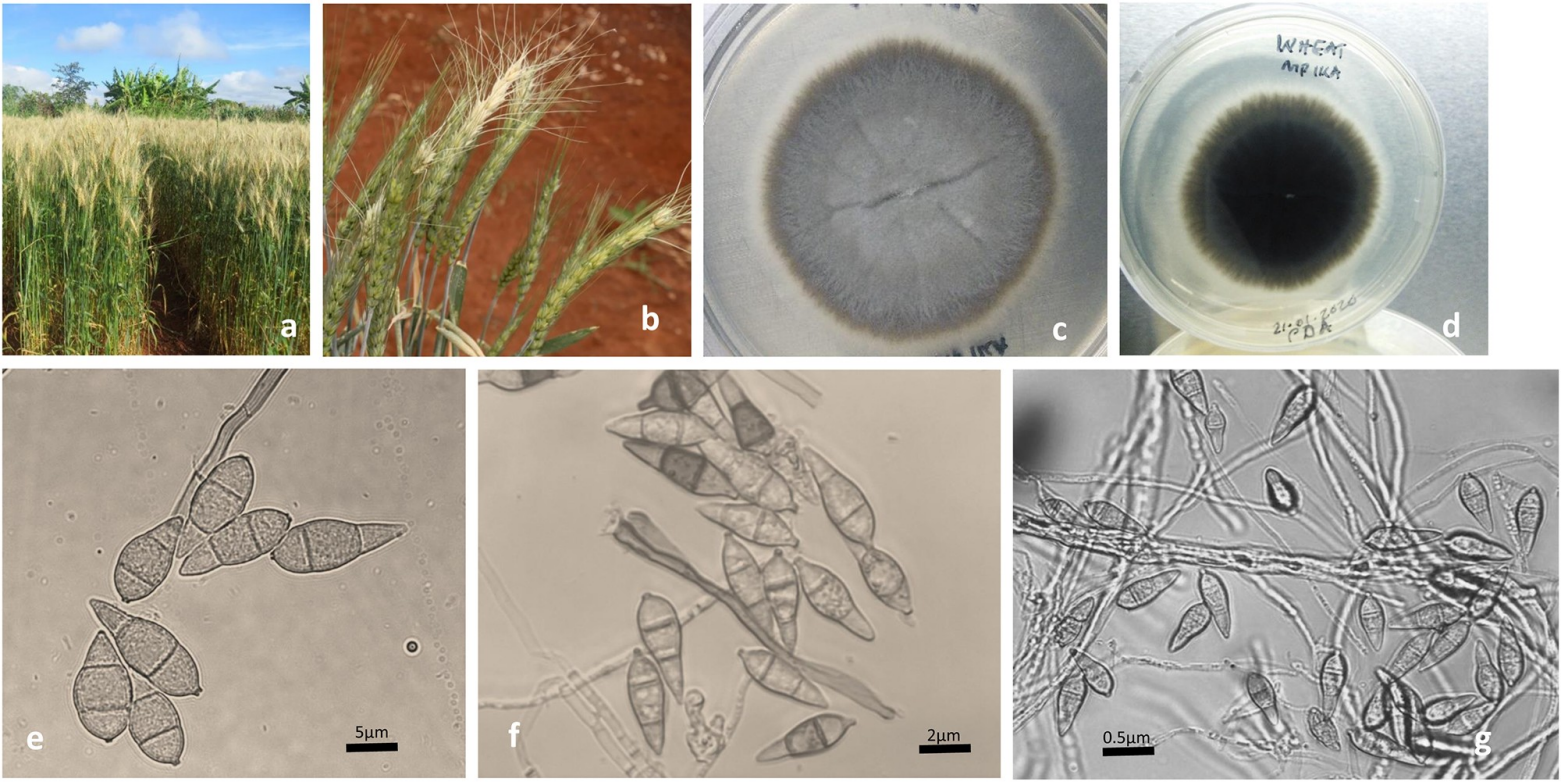
Wheat blast symptoms and spores isolated from symptomatic plants. A. Bleached heads of naturally infected plants of wheat variety Coucal. B. Wheat blast symptoms on inoculated plants in the entry restricted screenhouse. C. *Magnaporthe oryzae* pathotype *Triticum* (MoT) culture of isolate WS4. Grey mycelia at the bottom and white aerial mycelia can be observed. D. Color of mycelia on the reverse side of the same isolate looks dark. E, F and G. MoT Spores isolated from inoculated plants in the screenhouse. Spores were identified using a Motic BA310 digital microscope (Motic, Kowloon, Hong Kong).

All the fields inspected were highly infected with blast symptoms. Symptoms were observed on both leaves and spikes but only blast infected spikes were used for causal agent analysis. Disease incidence ranged from 50–100% and averaged 84.8%. Symptoms on most cultivars were widespread in both experimental and farmers’ fields, with Coucal being the most severely infected genotype with an averaged severity score of 5 (Fig 1A). Across the six fields, severity ranged from 2.5 to 5 with a mean value of 4.4 (Table 1).

**Table 1:** Incidence and severity of wheat blast recorded on wheat grown in experimental plots at Malashi and farmers’ fields at Mufubushi in Mpika district of Muchinga Province, Zambia.

Type of field (Exp/plot)	Variety	Source of seed planted	Blast incidence (%)	Severity
Experimental plot	Coucal	MMCRS	100	5
Farmers fields	Coucal	MMCRS	95	4.5
Experimental plot	RPWYT -1–35	ICARDA	80	4
Experimental plot	29TH HRWSN 8	CIMMYT	93	2.5
Experimental plot	29TH HRWSN 80	CIMMYT	50	3.5
Experimental plot	RPWYT -1-59	ICARDA	90	4.8
Experimental plot	HON 9	ICARDA	60	4.2
Experimental plot	HON 7	ICARDA	99	5
Experimental plot	37ESWYT 50	CIMMYT	80	5
Experimental plot	37ESWYT 94	CIMMYT	96	5
Experimental plot	Kwale	MMCRS	95	5

CIMMYT = International Maize and Wheat Improvement Center. ICARDA = International Centre for Agriculture Research in Dry Areas. MMCRS = Mount Makulu Central Research Station.

### Culture characteristic and spore morphology

Presentation of cultures on PDA varied but included several characteristic forms of mycelia such as moderate grey appearance distal to the central growth point, grey concentric rings, irregular margins, a white to light grey central erect sporulating aerial mycelia and dark color on the reverse side. The mycelium appeared septate (Fig 1F), whereas 2-septate hyaline pear-shaped conidia characteristic of *M*. *oryzae* (Fig 1E–1G) were observed on all the six cultures. These taxonomic characteristics of the fungus are however, not exclusive to the MoT thus, necessitating molecular characterization.

### Polymerase chain reaction

**Fig 2:**
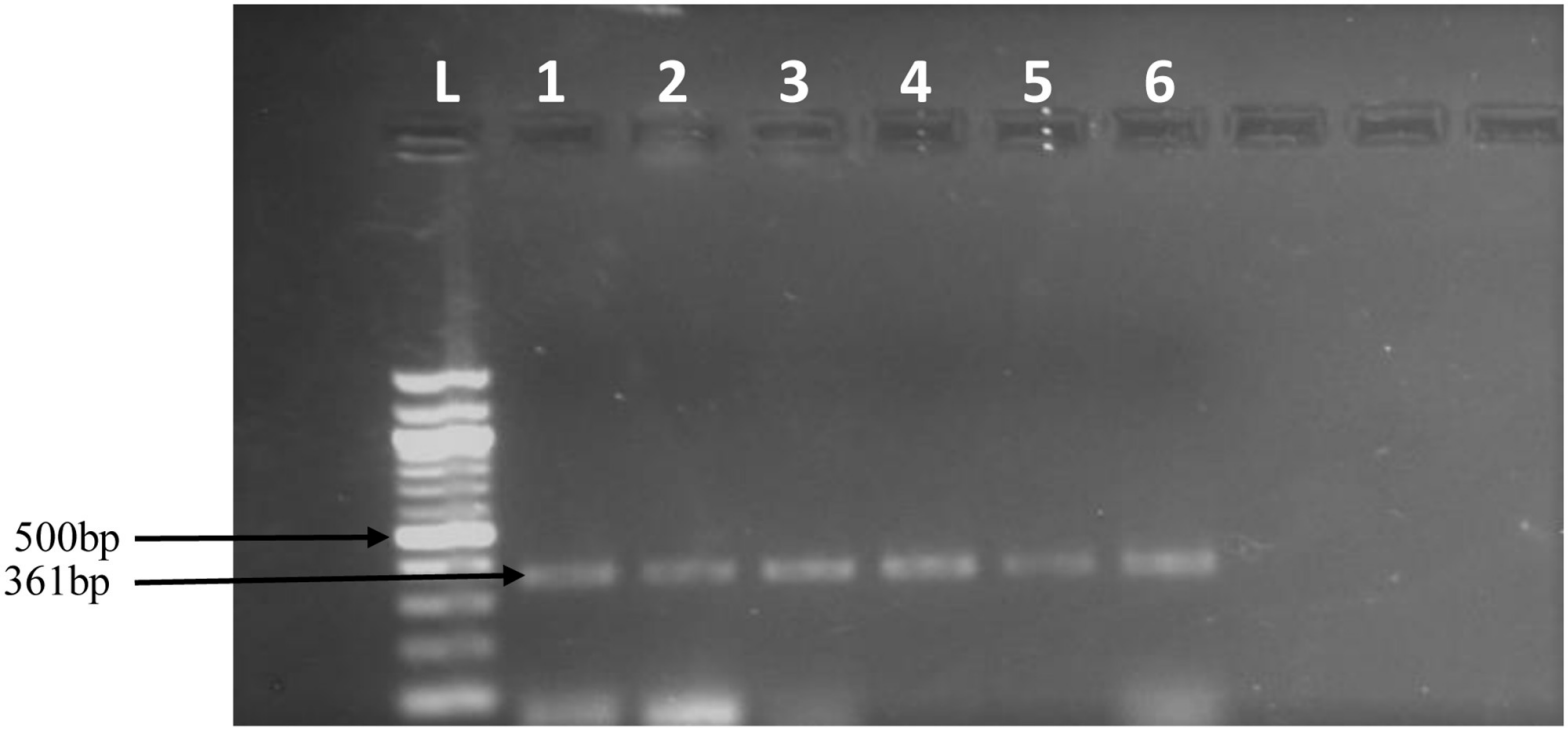
A. Amplified PCR fragments of expected bands using MoT3 oligonucleotide primers. Lane L is a 100 bp ladder. Lanes 1–6 are positive samples from the six symptomatic wheat head samples collected from both farmers’ fields and experimental fields in Mpika district, Zambia. B. Amplification of expected band sizes from two sub-isolates (WS4 and WS5) and two known MoT-negative (Rb3 –MoO and PL3.1-MoL) and positive (T25 and B2-MoT) samples with primers MoT3F/R and C17 F/R. As expected, there was no amplification from samples Rb3 (MoO) and PL3.1 (MoL). The analysis was performed at Ft. Detrick, MD, USA.

Preliminary screening of DNA from the six samples (WS1-WS6) with pathotype specific primers (MoT3F/R) [1] resulted in bands of the expected size (Fig 2A). Further PCR screening of sub-isolates Zambia1.2 (WS4) and Zambia 2.1 (WS5), two known MoT-negative samples (Rb3 and PL3.1) and two known MoT-positive samples (T25 and B2) with both MoT3F/R and C17 F/R [15] produced bands of the expected sizes (Fig 2B), thus confirming the identity of the *Magnaporthe* pathotype isolated in this study as MoT.

### Inoculation and symptom recovery

To further validate the pathotype of the identified *M*. *oryzae* isolates, wheat plants obtained from uninfected seed lots of all the six wheat genotypes included in the study were inoculated with cultures of morphobiometrically and PCR identified *M*. *oryzae* isolates that included the six MoT3 positive isolates. After 10 dpi, wheat blast symptoms were observed on a few of the plants from each pot (Fig 1C) and later all heads of all the inoculated plants were infected. Among the six wheat genotypes, RPWYT-1-35 was the first to show symptoms, whereas Coucal was the last. Symptoms were most severe in HRWSN80 suggesting its high susceptibility to the fungus. Pear-shaped hyaline conidia were recovered from inoculated samples (Fig 1E and 1F).

## Discussion

Our investigation concludes the first observation of the occurrence of wheat blast and its causal pathogen *Magnaporthe oryzae* pathotype *Triticum* (MoT) in Zambia and the foremost indication of its presence in Africa. Severe bleaching of wheat heads and other symptoms characteristic of wheat blast were common place in all wheat experimental fields and farmer-grown wheat fields in Mufubushi area located nearly 55 km from the experimental fields. Wheat blast is both wind-borne and seed-borne, with the former mainly responsible for inter-field disease spread and the latter for long-distance dispersal/introduction [20]. It is therefore plausible that the source of wheat seed planted at the two sites were already contaminated and inadvertently introduced in these areas. The other possibility is that, pathogen buildup could have taken place over a long time especially that Mpika is one of the earliest rain-fed wheat growing areas in Zambia. This can be seen in the high disease incidence and severity recorded in this study similar to fields reported with wheat blast in other studies [1, 13].

Detection and discrimination of *Magnaporthe spp*. causing blast in many species of Graminaceae has been challenging in the past due to close resemblances in the morphological characteristics and other species demarcation pathogenicity tests among different pathotypes. For example, *P*. *graminis-tritici* (*Pygt*) that was reported as separate from MoT (synonym abbreviation: *Pyricularia oryzae Triticum* [PoT]) [3, 21] was disputed in a recent study [22]. Although biological analysis of host-pathogen interactions and host range studies are critical in species demarcation, there still remains serious challenges in consensus definitive identification of the different host lineages of the ascomycetous fungus *P*. *oryzae*. In the past, multilocus housekeeping genes have been used to distinguish between closely related *P*. *graminis-tritici* (species demarcation disputed in Valent et al. 2019) and MoT (synonym abbreviation: *Pyricularia oryzae Triticum* [PoT]) [3]. However, housekeeping genes such as MPG1 (hydrophobin) seem conserved and therefore, although used in other studies for discriminating between isolates that have similar morphobiometrical characteristics, it is improbable that the gene can be a suitable differential marker. The recently discovered differential markers (MoT3 and C17F/R) that are exclusive to MoT among the *Magnaporthe* species [1, 15] were used in this study to detect the causal pathogens of wheat blast symptoms observed in both experimental wheat plots and farmers’ fields. PCR with MoT3F/R primers was performed independently at two laboratories giving the same results. Additional analysis of two sub-isolates with C17F/R primers conducted at USDA-ARS gave consistent positive results for MoT. Thus, results of the two analyses conducted at two independent laboratories coupled with the typical blast symptoms on artificially inoculated wheat plants confirm the hypothesis that wheat blast disease in Zambia is caused by MoT.

Whereas *M*. *oryzae* spp. have a worldwide distribution, high genetic diversity and host specialization, many recent studies have shown pathotypes previously known to infect wheat also infecting other members of the family *Poaceae* [2, 23]. This posed challenges in the past to base diagnosis on unilocus differential regions. Regardless, the MoT3 primers obtained through a rigorous analysis of a large number of blast fungal pathogens have been used before to detect the first ever isolate of MoT in Bangladesh [9]. Further elucidation of the whole genome reaffirmed the isolate identity and its lineage to the South American isolates [9]. Therefore, the amplification of size specific fragments from all the six samples and sub-isolates WS4 and WS5 coupled with the successful development of wheat blast symptoms on six inoculated wheat genotypes in pathogenicity tests is definitive of the pathotype.

In this study, the Zambian MoT isolates were pathogenic on six wheat genotypes grown in the screenhouse at Mount Makulu Central Research Station (MMCRS), causing typical spike blast symptoms. The response of the wheat genotypes in this study was not differential with all genotypes being similarly infected. In other studies, however, variations in symptom developments have been reported on different wheat cultivars and were attributed to cultivar resistance [3]. This implies that the tested wheat genotypes are all susceptible to wheat blast.

The impact of wheat blast on the yields of wheat have been well documented in many studies [10]. The logical step in combating the disease among farmers in Zambia is to address epidemiological factors supporting disease establishment. There are current efforts by the Zambia Agriculture Research Institute (ZARI) to promote rain-fed growing of wheat among smallholder farmers in Zambia. The positive impact of such efforts in Zambia will be reduced by the emergency of wheat blast disease coupled with other fungal diseases that have been reported in Zambia [16]. It is therefore important that measures to limit damage to wheat crops be instituted and collaborative research efforts started to prevent spread of the disease to new areas. Further studies aimed at understanding epidemiological factors supporting the spread of the causal fungus should be commenced.

In conclusion, this study provides evidence for the existence of a wheat blast-causing MoT fungus in the rain-fed wheat fields and experimental plots in Zambia. However, full genome characterization of the Zambian isolates is needed so as to understand the phylogenetic lineage and speciation of the fungus. It is also important to note that the pathogenicity tests in this study were limited to wheat samples that were observed with blast symptoms in the field. This study should be extended to other Poaceae plants so as to infer host range and pathotype differentiation. In the meantime, policy makers, plant quarantine and phytosanitary services, plant pathologists, wheat breeders and all stakeholders in the wheat value chain should collaborate in limiting further spread of the disease in rain-fed wheat fields. Resources should be allocated to regular monitoring and awareness among rain-fed wheat farmers.

## Supporting information

S1 Raw images (PDF)

S1 Data (XLSX)
