# Correction: Detection and characterization of fungus *(Magnaporthe oryzae pathotype Triticum)* causing wheat blast disease on rain-fed grown wheat (*Triticum aestivum* L.) in Zambia

**DOI:** 10.1371/journal.pone.0331932

**Published:** 2025-09-08

**Authors:** Batiseba Tembo, Rabson M. Mulenga, Suwilanji Sichilima, Kenneth K. M’siska, Moses Mwale, Patrick C. Chikoti, Pawan K. Singh, Xinyao He, Kerry F. Pedley, Gary L. Peterson, Ravi P. Singh, Hans J. Braun

Fig 2 is incorrect. Fig 2B does not appear. Please view Fig 2 here.

**Fig 2:**
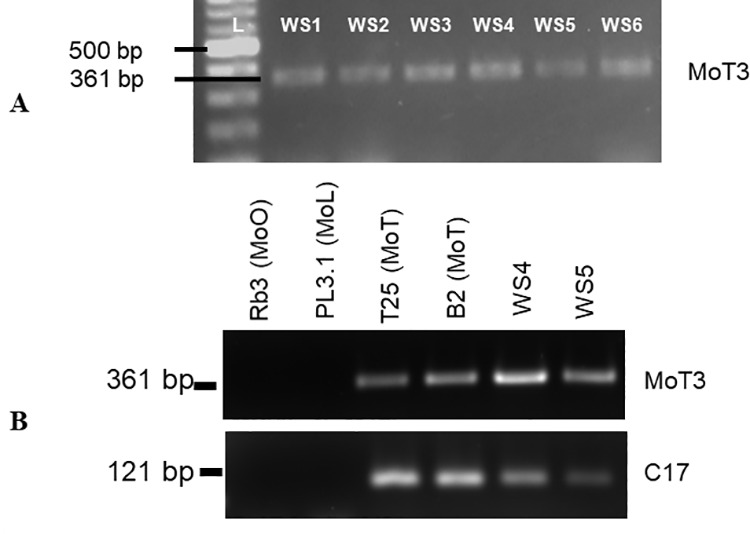
A. Amplified PCR fragments of expected bands using MoT3 oligonucleotide primers. Lane L is a 100 bp ladder. Lanes 1–6 are positive samples from the six symptomatic wheat head samples collected from both farmers’ fields and experimental fields in Mpika district, Zambia. B. Amplification of expected band sizes from two sub-isolates (WS4 and WS5) and two known MoT-negative (Rb3 –MoO and PL3.1-MoL) and positive (T25 and B2-MoT) samples with primers MoT3F/R and C17 F/R. As expected, there was no amplification from samples Rb3 (MoO) and PL3.1 (MoL). The analysis was performed at Ft. Detrick, MD, USA.
