# Supplementary material for: Detection and characterization of fungus (Magnaporthe oryzae pathotype Triticum) causing wheat blast disease on rain-fed grown wheat (Triticum aestivum L.) in Zambia
Source: PLoS One. 2020 Sep 21;15(9):e0238724. doi: 10.1371/journal.pone.0238724 (PMC7505438; doi:10.1371/journal.pone.0238724)
Supplement: S1 Raw images — (PDF) [file pone.0238724.s001.pdf]

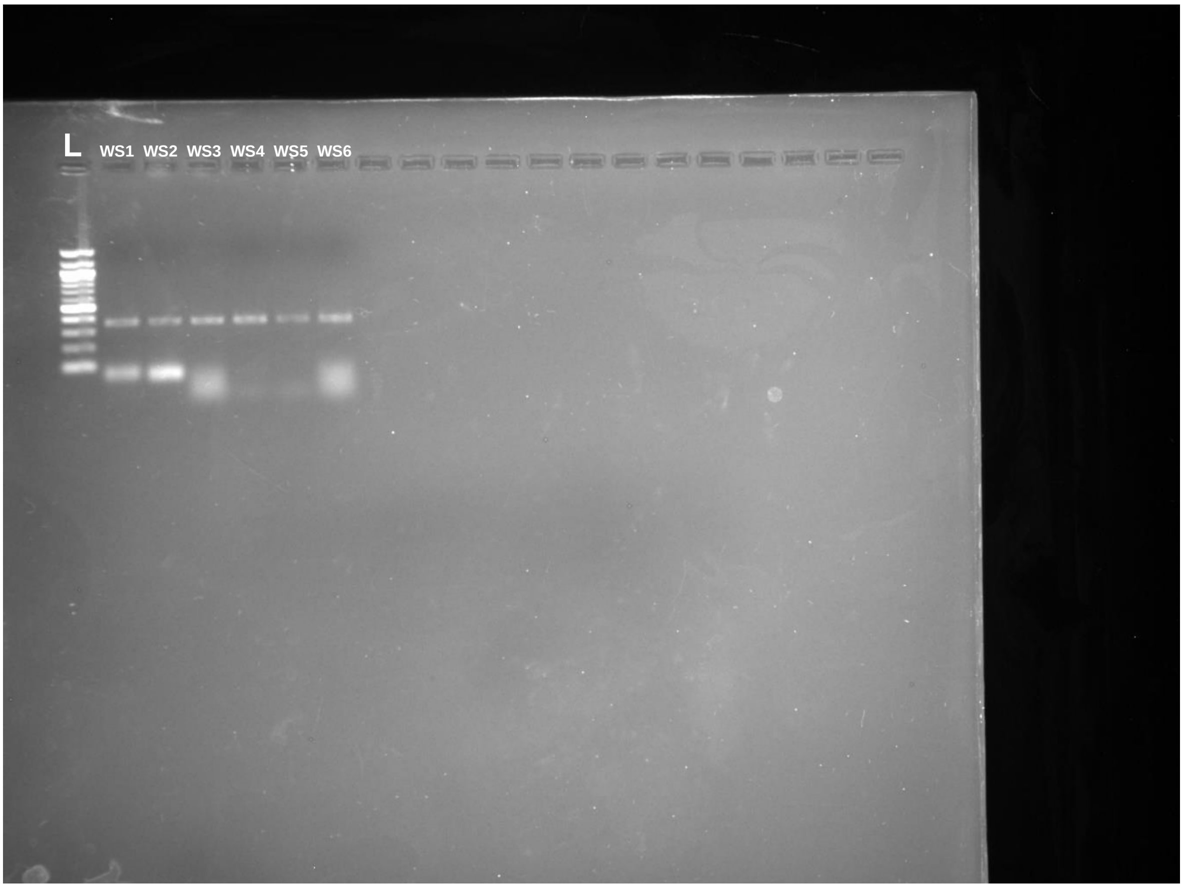

Preliminary amplification of PCR fragments of expected bands using MoT3 oligonucleotide primers. No positive controls were included. Lane L is a 100 bp ladder. Lanes WS1-6 are MoT positive samples from the six MoT infected wheat head samples collected from both farmers' fields and experimental fields in Mpika district, Zambia. The expected band size for the six positive bands is 361 bp. The gels were visualized using Gel Doc XR System (Bio-Rad Laboratories, Hercules, CA).

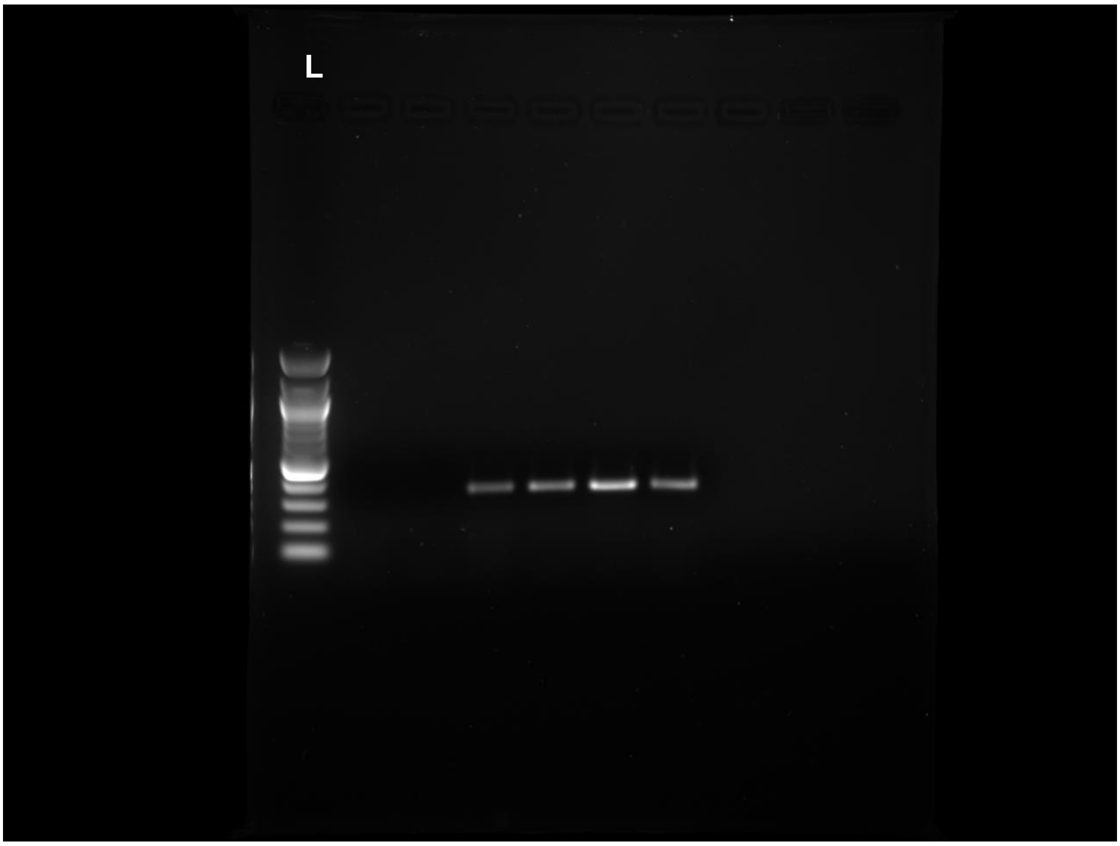

B1. L= 100 bp ladder. Lanes 1 and 2 are MoT-negative (Rb3 – MoO and PL3.1-MoL). Lanes 3 and 4 are positive (T25 and B2-MoT) and lanes 5 and 6 are two sub-isolates (WS4 and WS5). Amplification of expected band size (361bp) was achieved with primers MoT3F/R and visualized on Gel Doc XR System (Bio-Rad Laboratories, Hercules, CA)

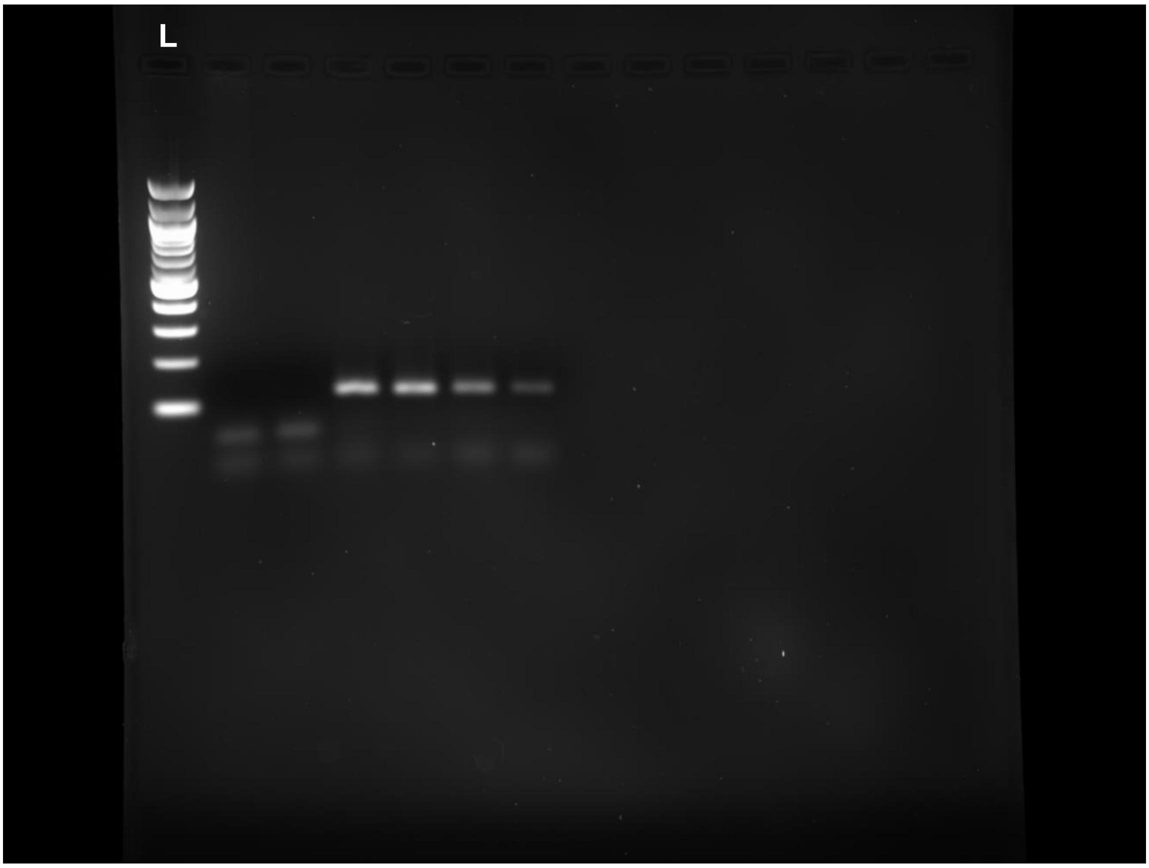

B2. L= 100 bp ladder. Lanes 1 and 2 are MoT-negative (Rb3 – MoO and PL3.1-MoL). Lanes 3 and 4 are positive (T25 and B2-MoT) and lanes 5 and 6 are two sub-isolates (WS4 and WS5). Amplification of expected band size (121bp)was achieved with primers C17 F/R and visualized using Gel Doc XR System (Bio-Rad Laboratories, Hercules, CA)
